# GLP‐1 agonist‐associated presentations to unscheduled care: An opportunistic pilot study

**DOI:** 10.1002/bcp.70335

**Published:** 2025-11-08

**Authors:** Oliver Thomas, James Michael Coulson

**Affiliations:** ^1^ Cardiff & Vale University Health Board, University Hospital of Wales, Heath Park Campus Cardiff UK; ^2^ All Wales Therapeutics & Toxicology Centre, Cardiff & Vale University Health Board, University Hospital, Llandough Penarth UK; ^3^ School of Medicine Cardiff University Cardiff UK

**Keywords:** GIP/GLP‐1, GLP‐1 receptor agonist, unscheduled care

## Abstract

The impact of GLP‐1 or GIP/GLP‐1 receptor agonist use on unscheduled care is not well‐described. We conducted a prospective, observational study using opportunistic screening by clinicians as a first step to understanding the effect of GLP‐1 or GIP/GLP‐1 agonists on Emergency Department presentations and to guide future studies. Data were collected from April to August 2025 at a single centre for any nontrauma adult patient who self‐reported GLP‐1 or GIP/GLP‐1 agonist exposure. Fifty‐six cases were identified, 51 reported tirzepatide use, three subcutaneous semaglutide, one oral semaglutide and one dulaglutide. In 79% of cases (CI95% 66% to 88%), the presenting features were due to GLP‐1 or agonist use. Presentations were greatest in the lowest and highest deprivation quintiles and a significant number had coexisting depression. There is a need for formal observational studies to assess the impact of GLP‐1 or GIP/GLP‐1 agonists on unscheduled care and explore these tentative associations.

**What is already known about this subject**

- The prevalence of GLP‐1 or GIP/GLP‐1 receptor agonist use within the population is believed to be increasing.
- The impact of GLP‐1 or GIP/GLP‐1 receptor agonist use on unscheduled care is unknown.

**What this study adds**

- Exposure to a GLP‐1 or GIP/GLP‐1 receptor agonist was considered the cause of the presenting features in 79% of Emergency Department attendees, who reported GLP‐1 or GIP/GLP‐1 receptor agonist use.
- The majority were females using tirzepatide from a private prescriber for weight management.
- Presentations were greatest in the lowest and highest deprivation quintiles and a significant number had coexisting depression.

## 1 INTRODUCTION

The glucagon‐like peptide 1 (GLP‐1) receptor agonists and the dual gastric inhibitory peptide (GIP)/GLP‐1 receptor agonist tirzepatide are indicated as second line treatments for type 2 diabetes mellitus and for weight management in obese (body mass index >30 kg.m^−2^) or overweight (body mass index >27 kg.m^−2^) patients with weight‐related comorbidities.^1^

Both GLP‐1 and GIP/GLP‐1 agonists act on the emetic centres of the central nervous system and commonly cause significant gastrointestinal side‐effects, including nausea and vomiting.^2^ An association between GLP‐1 agonists and non‐arteritic ischaemic optic neuropathy^3^ or an increased risk of acute pancreatitis^4^ remains controversial. The UK Medicines and Healthcare Regulatory Authority received notification of 42 deaths associated with GLP‐1 agonists and 18 deaths associated with GIP/GLP‐1 agonists.^5^

IQVIA, a market intelligence company, estimated there were over 1.5 million UK citizens receiving weight loss medications as of March 2025 and that a 56% increase in United Kingdom spending on private prescriptions from October 2024 to October 2025, was largely attributable to weight loss medications.^6^

The demand for GLP‐1 and GIP/GLP‐1 agonists is increasing in the obese and nonobese population,^7^ yet supply through state healthcare is limited. Private providers offer GLP‐1 or GIP/GLP‐1 agonists following an initial consultation, be that face‐to‐face or remotely. Patients can also acquire these medications illegitimately through online marketplaces or nonregulated sellers, which risks the possibly of contamination and misrepresentation.^8^

The impact of GLP‐1 or GIP/GLP‐1 agonist exposure in a significant proportion of the population is associated with safety risks in patients undergoing endoscopy^9^ or general anaesthesia.^10^ The impact on GLP‐1 or GIP/GLP‐1 agonists on unscheduled care is not well‐described. We conducted a prospective, single‐centre, observational study using opportunistic screening by clinicians as a first step to understanding the effect of GLP‐1 or GIP/GLP‐1 agonists on Emergency Department presentations and to guide future studies.

## 2 METHODS

Data were collected over a 120‐day period from April to August 2025 at a tertiary centre providing care to a population of approximately 500 000 individuals. Emergency Department clinicians were encouraged to complete an electronic questionnaire for any nontrauma adult (age >18 years) patient with GLP‐1 or GIP/GLP‐1 agonist exposure, regardless of their indication for use, or whether their presentation was perceived to be because of GLP‐1 or GIP/GLP‐1 agonist use. Identification of GLP‐1 or GIP/GLP‐1 agonist exposure relied on routine medication history‐taking or identification from electronic medical records by the assessing clinician. Data collection was opportunistic and relied on submission of the electronic questionnaire; there was no systematic screening of patients who attended the Emergency Department.

Data were collected on patient demographics; presenting complaint and clinical symptoms; clinical diagnosis at discharge; type, dosage and recent dose changes of GLP‐1 or GIP/GLP‐1 agonist. Clinicians were also asked if, in their professional judgement, the patient's presentation was a result of GLP‐1 or GIP/GLP‐1 agonist exposure. Electronic records were screened for coexisting medical conditions, including obesity, defined as a body mass index >30 kg.m^−2^. The Welsh Index of Multiple Deprivation (WIMD) Score was determined from the patient's postcode using the Data Cymru website.^11^ Data were collected as part of a service evaluation of routinely collected clinical information. As such, explicit consent was not required for inclusion.

Data were analysed using Excel (Office 365 Subscription, Microsoft Corporation, San Diego, CA, USA) and GraphPad Prism (GraphPad Software LLC, San Diego, CA, USA). Data were presented as counts and percentages or median and interquartile range (IQR). Proportions were expressed as a decimal and 95% confidence interval (CI_95%_). Chi‐squared or Fishers exact test were used to test independence of categories. The Kruskal–Wallis test was used to compare nonparametric data. An alpha less than 0.05 was considered significant.

Data were collected in accordance with the relevant University Health Board policies, as part of a service evaluation and was approved by the Quality and Safety lead for Emergency Medicine.

Key protein targets and ligand in this article are hyperlinked to corresponding entries in http://www.guidetopharmacology.org and are permanently archived in the Concise Guide to PHARMACOLOGY 2023/24.^12^

## 3 RESULTS

**FIGURE 1:**
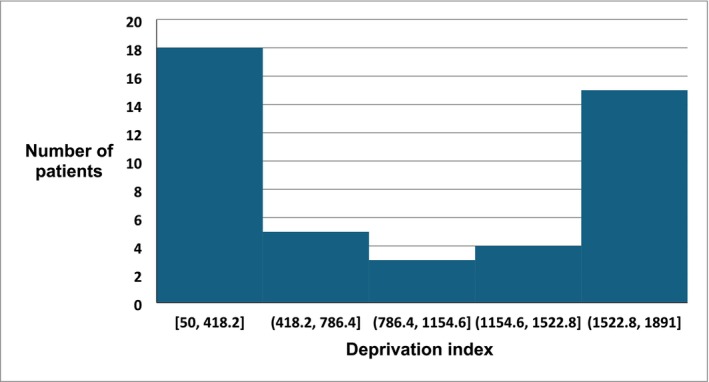
The number of patients in each quintile of the Welsh Index of Multiple Deprivation Score, as determined by postcode.

Fifty‐six Emergency Department attendances met the inclusion criteria over the 120‐day study period. Of these, two attendances were by the same patient but with different symptoms. The median age was 38 years (IQR 28.75–51.25 years), with an age range from 18 to 88 years. Forty‐four patients (79%) were female, *p* < 0.0001. The median WIMD Score was 785 (IQR 181–1657), ranged from 50 to 1891. The number of cases in each WIMD quintile was not evenly distributed across the quintiles, Figure 1, *p* < 0.00001. Eighteen cases (32%) were in the lowest quintile and 15 (27%) in the highest.

Twenty‐six patients (46%) had a diagnosis of depression; 14 (25%) had a body mass index >30 kg.m^−2^; 10 (18%) cardiovascular disease, including hypertension or atrial fibrillation; four (7%) had gallstones; four (7%) Type 2 diabetes mellitus and three (5%) were prediabetic.

Fifty‐one patients (91%) reported using subcutaneous tirzepatide (Mounjaro), three subcutaneous semaglutide (Ozempic/Wegovy), one oral semaglutide (Rybelsus) and one dulaglutide (Trulicity). The dose of tirzepatide was recorded in 45 cases, and the median weekly subcutaneous dose was 5 mg (IQR 2.5–8.75 mg), range 2.5 to 15 mg. The weekly doses of subcutaneous semaglutide ranged from 1 to 1.25 mg, the daily oral semaglutide dose was 7 mg, equivalent to a weekly semaglutide dose of 0.25 mg, and the weekly subcutaneous dulaglutide dose was 3 mg.

Nineteen patients reported a dose increase within 2 weeks of presentation and 35 reported no change in dose within 2 weeks. Two patients reported drug discontinuation 1.5 and 4 weeks prior to presentation, respectively.

The median time between starting a GLP‐1 or GIP/GLP‐1 agonist and attendance to hospital was 45 days (IQR 20–123 days), range 0 to 422 days. The median time to hospital admission did not differ between GLP‐1 or GIP/GLP‐1 agonists, *p* = 0.1259.

Fifty‐two patients (93%) were using a GLP‐1 or GIP/GLP‐1 agonist for weight management and four for type 2 diabetes mellitus (T2DM) (7%). Eighty‐four percent of patients using a GLP‐1 or GIP/GLP‐1 agonist for weight management were female compared with 50% for T2DM, *p* = 0.1571. The median age for patients using a GLP‐1 or GIP/GLP‐1 agonist for weight management was 38 years (IQR 29–48.5 years) compared with 69.5 years (IQR 36.75–76.75 years) using a GLP‐1 or GIP/GLP‐1 agonist for T2DM, p = 0.06.

Overall, 77% of patients were prescribed a GLP‐1 or GIP/GLP‐1 agonist by a private prescriber, CI95% 64% to 87%. Forty‐one patients using tirzepatide reported that they were prescribed it by a private prescriber, 36 of which were prescribed via the internet. Four were prescribed tirzepatide by primary care, two by secondary care and four obtained tirzepatide without a prescription. Semaglutide was prescribed by a private internet prescriber in two cases, by secondary care in one case and without a prescription in one case. Dulaglutide was prescribed by primary care.

Clinical features are detailed in Table 1. Forty‐four patients (79%) reported gastrointestinal features. These included 39 (70%) with abdominal pain, 36 (64%) nausea or vomiting, 11 (20%) diarrhoea, eight (14%) anorexia, six (11%) cholecystitis, five (9%) constipation and two (4%) acute pancreatitis. Nineteen (43%) reported constitutional symptoms, including 12 (21%) with dehydration, 12 (21%) fatigue, four (7%) weight loss and one (2%) fever.

**TABLE 1:** Clinical feature categories and clinical features.

		*n*	%
Gastrointestinal		44	79
	Abdominal pain	39	70
	N&V	36	64
	Diarrhoea	11	20
	Anorexia	8	14
	Cholecystitis	6	11
	Constipation	5	9
	Acute pancreatitis	2	4
**Constitutional**		**19**	**43**
	Dehydration	12	21
	Fatigue	12	21
	Weight loss	4	7
	Fever	1	2
**Neurological**		**8**	**14**
	Headache	6	11
	Dizziness	6	11
	Seizure	2	4
**Cardiovascular**		**6**	**11**
	Chest pain	6	11
	Palpitations	2	4
**Metabolic**		**6**	**11**
	Electrolyte abnormalities	4	7
	DKA	1	2
	Hypoglycaemia	1	2
**Dermatological**	**Skin rash**	**1**	**2**

Eight (14%) reported neurological features, including six (11%) with headache, six (11%) dizziness and two (4%) seizures. Six (11%) reported cardiovascular features of chest pain in six (11%) and two (4%) palpitations. Six (11%) had metabolic features, including four (7%) with electrolyte disturbances, one (2%) with diabetic ketoacidosis and 1 (2%) hypoglycaemia. One (2%) reported a skin rash.

Forty‐three patients who reported using tirzepatide had gastrointestinal features at presentation, five had cardiac features, two had constitutional features, and one had neurological features. Three of the five patients who reported GLP‐1 agonist use had gastrointestinal features at presentation, one had constitutional features, and one had neurological features. The frequency of gastrointestinal features compared to other feature categories was independent of tirzepatide verses GLP‐1 agonist exposure, *p* = 0.0631.

In 33 cases (59%), the assessing clinician considered that the presentation was due to GLP‐1 or GIP/GLP‐1 agonist exposure, and in 11 (20%), the assessing clinician considered that the presentation was possibly due to GLP‐1 or GIP/GLP‐1 agonist exposure. Overall, in 79% (CI95% 66%–88%) of cases clinicians considered or suspected that the presentation was due to GLP‐1 or GIP/GLP‐1 agonist exposure. The attribution of a causal relationship was independent of class of clinical feature, *p* = 0.2476.

Forty‐two (75%) patients were discharged direct from the Emergency Department, with follow‐up arrangements made for five. Nine (16%) were admitted to General Surgery and five (9%) to Medicine.

## 4 DISCUSSION

Opportunistic screening of Emergency Department attendees identified 56 patients exposed to GLP‐1 or GIP/GLP‐1 agonists. The vast majority were female, who reported using tirzepatide prescribed by a private prescriber for weight management. In 79% of cases, the assessing clinician considered that GLP‐1 or GIP/GLP‐1 agonist exposure caused or might have caused the presenting features. There was a ‘U‐shaped’ relationship between postcode‐derived deprivation score, with the majority of in the lowest or highest quintiles.

The prevalence of GLP‐1 or GIP/GLP‐1 agonist use within the United Kingdom is unknown since most prescribing is probably through private providers. Screening of National Health Service prescription databases are likely to underestimate the true prevalence and are unlikely to represent the full population using GLP‐1 or GIP/GLP‐1 agonists. There is no reliable estimate of the socioeconomic distribution of GLP‐1 or GIP/GLP‐1 agonists exposure or side‐effect risk.

This study observed that almost half of patients had a coexisting diagnosis of depression. GLP‐1 agonist use does not appear to be associated with an increased risk of depression.^13^ The prevalence of depression increases with body mass index and is greater in females compared with males^14^; however, only 25% of our study had a body mass index >30 kg.m^−2^ documented in their medical records, suggesting that the association is not fully explained by a bidirectional relationship. GLP‐1 agonists are less effective in patients co‐prescribed antidepressants.^15^ The mechanisms for this are unclear, but it is possible that incidence of GLP‐1 or GIP/GLP‐1 agonist side‐effects is increased in those with depression or receiving antidepressants.

The reported clinical features reported were predominantly gastrointestinal and occurred across the GLP‐1 or GIP/GLP‐1 agonist dosing range. There was no difference in the frequency of gastrointestinal features compared to other feature categories between tirzepatide compared to the GLP‐1 agonists, consistent with previous studies.^16^

In 79% of cases, the assessing clinician considered that GLP‐1 or GIP/GLP‐1 agonist exposure caused or might have caused the presenting features. At a clinical level, there is a high probability that prescription of a GLP‐1 or GIP/GLP‐1 agonist will not be recorded on the patient's medical records. It is recognized that patients may not consider weight management drugs to be medications and may not declare their use without direct questioning.^10^ This highlights the importance of a thorough medication history as part of diagnostic synthesis.

Our study has several limitations. Patient identification was opportunistic at a single point of admission to a single site and relied on patients self‐reporting in response to clinician screening; it was not possible to estimate the prevalence of cases presenting to the Emergency Department with a history of GLP‐1 or GLP‐1/GIP agonist exposure or to determine odds ratios for identified associations. There was also a strong risk of selection bias; however, the independence of clinical features from the attribution of a causal association by clinicians suggests that the risk of confirmatory bias was not high. We did not undertake any analytical confirmation of GLP‐1 or GIP/GLP‐1 agonist exposure; however, the clinical features reported generally confirmed to recognized GLP‐1 or GIP/GLP‐1 agonist side‐effects.

This opportunistic pilot study identified that the majority of patients presenting to a single Emergency Department who reported GLP‐1 or GIP/GLP‐1 agonist use were females using tirzepatide for weight management from private prescribers. In 79% of cases, the presenting features were due to GLP‐1 or GIP/GLP‐1 agonist use. Presentations were greatest in the lowest and highest deprivation quintiles and a significant number had coexisting depression. There is a need for formal observational studies to rigorously assess the true impact of GLP‐1 or GIP/GLP‐1 agonist exposure on unscheduled care and explore these tentative associations.

## AUTHOR CONTRIBUTIONS

Both authors contributed equally to the study and the manuscript.

## CONFLICT OF INTEREST STATEMENT

The authors declare no conflicts of interest.

## Data Availability

The data that support the findings of this study are available from the corresponding author upon reasonable request.
